# Primary intraspinal neuroendocrine tumor: A case report and literature review

**DOI:** 10.1097/MD.0000000000039196

**Published:** 2024-08-09

**Authors:** Li Dai, Ming-Ju Zou, Ren-Li Liao, Bing-Ran Zhang, Zhi-Qiang Ma, Ming-Wei Liu

**Affiliations:** a Department of Endocrinology, The Second People’s Hospital of Jiangjin District, Chongqing, China; b Department of Orthopedics, People’s Hospital of Dali Bai Autonomous Prefecture, Dali, Yunnan, China; c Department of Emergency, The First Affiliated Hospital of Kunming Medical University, Kunming, Yunnan, China; d Department of Clinical Laboratory, People’s Hospital of Dali Bai Autonomous Prefecture, Dali, Yunnan, China; e Department of Emergency, People’s Hospital of Dali Bai Autonomous Prefecture, Dali, Yunnan, China.

**Keywords:** case report, diagnosis, limb numbness, limb weakness, neuroendocrine tumor, treatments

## Abstract

**Rationale:**

Neuroendocrine tumors (NET) refer to a group of uncommon tumors arising in the neuroendocrine system. Most NETs occur in the digestive tract and bronchi but are rare in the central nervous system, especially in the spinal canal. NET in the central nervous system mainly metastasize from other systems, with non-specific clinical symptoms. In this study, we report the diagnosis and treatment of intraspinal NET to provide clinical guidance as well as to avoid misdiagnosis and missed diagnosis.

**Patient concerns:**

A 59-year-old male patient, presented with recurrent right lower limb pain for half a year, accompanied by numbness and weakness for 4 months and aggravation for 2 months. Lumbar spine magnetic resonance imaging (MRI) revealed a space-occupying lesion in the spinal canal. The diagnosis of primary intraspinal NET was confirmed by topathological examination.

**Diagnosis:**

Primary intraspinal NET tumor.

**Interventions:**

Surgical resection.

**Outcomes:**

Significant improvements in right lower limb pain, numbness, and weakness were observed, and lumbar spine MRI was performed again to dynamically observe the changes in intraspinal NET.

**Conclusions:**

Surgical resection may be an effective treatment for intraspinal NETs.

**Lessons:**

Intraspinal NETs are relatively rare and mostly manifest as limb numbness, weakness, and pain. Due to its nonspecific clinical symptoms, intraspinal NETs are easily misdiagnosed as lumbar disc herniation with radiculopathy and lumbar spondylolisthesis. Therefore, in patients with long-term symptoms, in addition to common lumbar neuromuscular diseases, lumbar MRI should be performed promptly to exclude the possibility of lumbar NETs.

## 1. Introduction

Neuroendocrine tumors (NETs) are rare heterogeneous tumors arising from peptidergic neurons and neuroendocrine cells with neuroendocrine differentiation and neuroendocrine marker expression. NETs are extremely widely distributed in the human body, such as pure endocrine organs (e.g., anterior pituitary gland, parathyroid gland, thyroid gland, and adrenal gland), pure neural structures (e.g., paraspinal sympathetic chain and parasympathetic ganglion), and multiple systemic organs with diffuse neuroendocrine cell systems (e.g., skin, upper respiratory tract, lung, breast, digestive, urinary, and reproductive organ systems).^[1]^ According to carcinoid tumor patients registered from 1950 to 1999 in the Surveillance, Epidemiology, and End Results database, NETs in the gastrointestinal tract and bronchopulmonary system accounted for 66.9% and 24.5% of all sites, respectively.^[2]^ In addition, the primary sites of NETs vary in sex and race. Specifically, the common sites of NETs in female patients are the lungs, stomach, appendix, and cecum, whereas, in male patients, they are the thymus, duodenum, pancreas, jejunoileum, and rectum.^[3]^

Because NETs rarely occur in the spinal canal,^[3]^ nonspecific clinical symptoms such as limb pain, weakness, numbness, diagnosis, and missed diagnosis can easily occur in clinical settings. In this case report, we investigated the diagnosis and treatment of intraspinal NET to provide clinical guidance, as well as to avoid misdiagnosis and missed diagnosis.

## 2. Case report

### 2.1. Ethics approval and consent to participate

Informed written consent was obtained from the patient for publication of this case report and accompanying images.

This study was reviewed and approved by the local ethics committee of the First Affiliated Hospital of the Kunming Medical University. The procedures were performed in accordance with the Helsinki Declaration of 1975, revised in 2000.

### 2.2. Case presentations

A 67-year-old male patient visited the local hospital due to recurrent right lower limb pain for half a year, accompanied by numbness and weakness for 4 months and aggravation for 2 months. The pain was paroxysmal for approximately 10 minutes each time. The patient was diagnosed with lumbar disc herniation (LDH) with radiculopathy and lumbar spondylolisthesis, and underwent minimally invasive ablation of LDH. Symptoms did not improve significantly after surgery and were slightly alleviated after acupuncture. However, the patient still experienced numbness and discomfort below the bilateral buttocks, with weakness of both lower limbs and inability to walk. Thus, he was admitted to the Department of Neurology at the First Affiliated Hospital of Kunming Medical University for further treatment.

### 2.3. Medical history

The patient had hypertension for 4 years and did not take antihypertensive drugs regularly. In addition, he had diabetes for 5 years and self-administered sitagliptin metformin for 2 years, followed by spontaneous discontinuation and poor glycemic control. The patient had a lumbar fracture due to a car accident in 1995 and underwent lumbar traction. Pituitary tumor resection and minimally invasive ablation of LDH were performed in 2008 and 2023, respectively. No history of blood transfusions was reported. Allergies included sulfonamide and penicillin, and the history of vaccination was unknown.

### 2.4. Physical examination

The patient could cooperate with the physical examinations: clear consciousness, Glasgow Coma Scale score 15 points, fluent speech, and normal abilities in orientation, calculation and memory; soft neck, no refusal and resistance, negative Kernig sign and Brudzinski sign; equal and round pupils bilaterally with diameter of 3 mm, direct and indirect light reflexes, all directions of both eyes were active, no diplopia, normal visual field, symmetrical bilateral frontal lines; symmetrical bilateral nasolabial folds, no deviation of mouth, no air leakage from the drumsticks, strong chewing, centered tongue extension, fair bilateral soft palate elevation, centered uvula, bilateral gag reflex (+ +), strong head up and neck rotation; decreased muscle volume of both lower limbs (mainly right lower limb), grade 4 muscle strength for left lower limb, grade 3 muscle strength for right lower limb. Decreased muscle tone in both lower limbs, no tendon reflexes, or pain allergy. Normal limb sensory testing (warmth, pressure, touch, vibration, pain, and sensation). Negative Romberg sign, and alternate motion revealed bilateral symmetry without clumsiness. Positive results for both lower limbs in straight-leg raise testing and Patrick (FABERE) test; normal nutrition of skin, hair, and nails; normal urine and defecation; unremarkable skin scratches, and grade I in the water swallow test.

### 2.5. Laboratory test

White blood cell count6.34 × 10^9^/L, neutrophils 59.6%, lymphocytes 30.4%, eosinophils 3.20%, red blood cell count 4.82 × 10^12^/L, hemoglobin 134g/L, and platelet count 302 × 10^9^/L. Sodium 143.60 mmol/L, chlorine 105.40 mmol/L, alanine aminotransferase 67.00 IU/L, aspartate aminotransferase 21.43.00 IU/L, AST/alanine aminotransferase 1.47, creatinine 76.30 µmol/L, urea nitrogen 4.24 µmol/L. Blood glucose 7.2 mmol/L, serum fructosamine 297.5 mmol/L, glycosylated hemoglobin 7.3%, β-hydroxybutyrate 0.02 mmol/L, anti-alkaline hemoglobin, 0.03%; creatine phosphokinase 60.70 IU/L, creatine phosphokinase MB isoenzyme 12.10 IU/L, lactate dehydrogenase, 201 IU/L. The following tests yielded negative results: fungal smear, bacterial Gram staining, immunoglobulin and complement, Fehling’s test, Witting reaction, tumor markers, urine routine, systemic lupus erythematosus, rheumatoid associated antibodies, anticardiolipin antibodies, and antineutrophil cytoplasmic antibodies.

### 2.6. Imaging results

**Figure 1.**
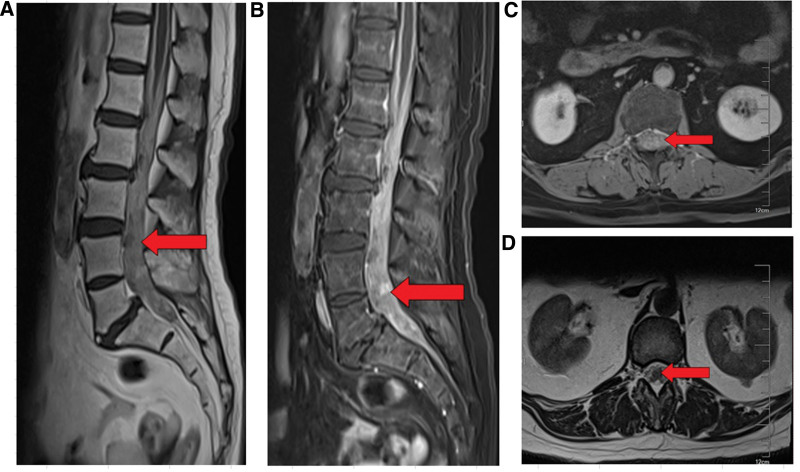
MRI of the lumbar spine. (A–D) Lumbar spine MRI. MRI = magnetic resonance imaging.

According to lumbar spine magnetic resonance imaging (MRI), acylindrical iso-T1 and iso-slightly long T2 soft tissue masses in the spinal canal of the L3-S1 plane almost occupied the whole spinal canal, and heterogeneous and moderate enhancement was observed, with a maximum axial diameter of approximately 21 × 16 mm (Fig. 1). The lesion involved the cauda equina upward along the spinal canal, and the cervical thoracolumbar meninges were widely unevenly thickened and significantly enhanced, with a length of approximately 10 mm. The local spinal cord was compressed and the signal on T2W1 was slightly enhanced (Fig. 1). The dura mater and leptomeninges were widely thickened and significantly enhanced in the enhanced range of the cervical vertebra, especially the leptomeninges (Fig. 1).Small nodular enhancement lesions were observed in the anterosuperior border of the pontine, and the vertebral bodies adjacent to the lesion were not destroyed (Fig. 1).

### 2.7. Pathology and immunohistochemistry

**Figure 2.**
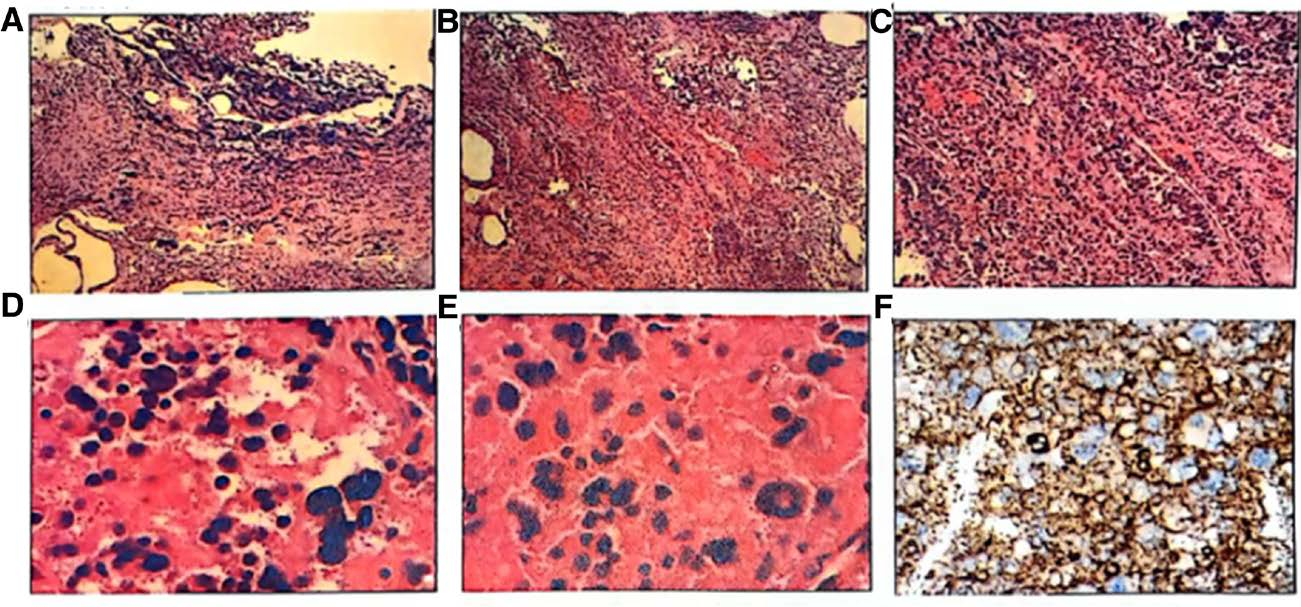
Histopathological assay of the spinal space-occupying lesions and ligamentum flavum. (A–F) Histopathology results of spinal space-occupying lesions.

Pathological examination noted T2-3 and L1-S1 intraspinal space. Surgical resection removed 4 pieces of grayish-red aplastic tissue with a total volume of 2.0 cm × 2.0 cm × 1.0 cm, and the cut surface was grayish red, solid, soft, and full mass (Fig. 2). Intraspinal ligamentum flavum surgery removed a grayish-yellow grayish-red aplastic tissue with a total volume of 3.0 cm × 1.5 cm × 1.0 cm, and the cut surface was grayish-yellow and grayish-brown, solid, and medium in quality (Fig. 2). Microscopically, the tumor cells were arranged in nests or irregular adenoids, the cell morphology was relatively consistent, the nuclei were round or oval, the chromatin was delicate, and the mitotic figures were not easily visible (Fig. 2).

Immunohistochemistry: ALK (−), Bcl-2 (+), CD10 (−), CD138 (−), CD20 (−), CD3 (−), CD38 (−), CD45RO (−), CD79a (−), CD99 (+), CydinD (−), GFAP (−), Ki67 (10% +), NKX2.2 (−), Pan-CK (paranuclear dot +), S-100 (−), SOX10 (−), Vim (−), CD117 (−), CD34 (vascular +), CD68 (−), LCA (−), Mpo (−), OLIG-2 (−), CD56 (+), CgA (+), CK8/18 (paranuclear dot +), EMA (−), ER (−), GATA3 (focal −), INSM (+), PTH (−), Syn (+), Syn (+), TTF-1 (−), CDX-2 (−).

### 2.8. Diagnosis and treatment

**Figure 3.**
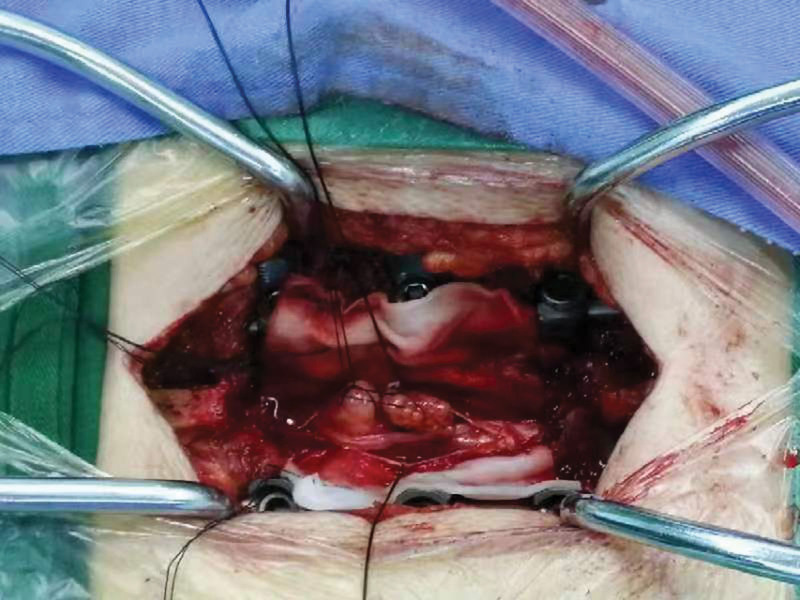
Surgical resection of lumbar spine tumor.

These results verified the diagnosis of primary intraspinal NET and redundant nerve root syndrome of the cauda equina. After 1 week of treatment, the patient underwent surgical resection (Fig. 3) and was discharged for observation.

### 2.9. Follow-up

We conducted a follow-up visit 1 month after the patient’s discharge, and the symptoms of limb numbness, weakness, and pain were significantly relieved. Reexamination of the lumbar spine MRI was performed to dynamically observe the changes in intraspinal NET.

## 3. Discussion

Primary intraspinal tumors refer to the collective term of both primary tumors and metastatic tumors growing in the spinal cord itself and in the tissue structures adjacent to the spinal canal and spinal cord (e.g., nerve roots, dura mater, intraspinal adipose tissue, blood vessels, etc.), mainly manifested as radicular pain, sensory disturbances and dyskinesia. Intraspinal tumors are common and diseases in spinal orthopedics and neurosurgery, accounting for 10% to 15% of neurological tumors.^[4]^ Clinical manifestations of intraspinal tumors are closely related to their growth site and size in the spinal canal. Since early symptoms are nonspecific and atypical, intraspinal tumors are difficult to differentiate from maternal diseases.^[5]^ To be specific, both LDH and intraspinal tumors can cause lower back and leg pain, leading to delayed treatment.^[3]^ In addition, if intraspinal metastases invade the lumbar spinal canal, they can also result in low back pain and lower limb neurological symptoms and signs and are often misdiagnosed as LDH.^[4]^ Just to the patient in this study, the repeated wrong diagnosis of LDH radiculopathy and lumbar spondylolisthesis during the course of the disease led to mistreatment.

NET have a predilection for the digestive tract and bronchi, and other sites include the bile duct, pancreas, ovary, testis, thymus, thyroid gland, breast, and parotid gland.^[1]^ Although the central nervous system is not a common site for NET,^[6]^ literatures has reported that most intraspinal NET can metastasize from other sites, so intraspinal NET can be divided into either primary or metastatic. Metastatic intraspinal NET mainly occur epidurally and rarely occur in subdural or intramedullary metastases.^[7]^ Tsimpas et al^[8]^ suggested that no more than 5% of metastatic intraspinal NET develop in the subdural space. In this case report, the low-grade intraspinal subdural NET was closely related to the filum terminale, and because head and chest computed tomography revealed no other systemic tumor lesions, this case was more likely to be a primary NET. In addition to symptoms caused by primary intraspinal tumors, symptoms of carcinoid syndrome may also occur, such as facial flushing, nausea, vomiting, diarrhea, asthma, cardiac symptoms, blood pressure fluctuations, and pigmentation.^[9]^ The patient had no symptoms of carcinoid syndrome except numbness and pain in the extremities.

Imaging examinations play a dominant role in the diagnosis of intraspinal NET.^[10]^ MRI findings for intraspinal NET can be diverse, mostly extramedullary subdural lesions showing low or isointensity on T1WI and isointensity or slightly high on T2WI, with ring enhancement around the lesion after enhancement and mild internal enhancement. However, it can also exhibit heterogeneous enhancements. Since most intraspinal NET are metastatic, cranial and thoracoabdominal computed tomography is required to rule out the possibility of metastasis.^[11]^ Despite the importance of imaging in the diagnosis of NET, it needs to be differentiated from schwannoma and meningioma, and MRI is generally preferred in clinical practice. However, the diagnosis depends on pathology because MRI findings of intraspinal NET are nonspecific,^[12]^ and some imaging features do not meet the typical characteristics of common tumors; for example, the ring enhancement in this case report should be combined with the relevant clinical symptoms before considering the possibility of NET. The MRI results of our patient suggested a cylindrical iso-T1 and iso-slightly long T2 soft tissue mass in the spinal canal of the L3-S1 plane; however, NET could not be confirmed.

This patient had a confirmed diagnosis of low-grade NET based on a 10% positiveKi-67 index in the pathology and previous literature.^[13]^ At present, surgical resection remains the most important treatment for intraspinal NET. Even if the tumor cannot be completely removed by surgery, it can relieve the postoperative tumor burden and provide favorable conditions for other postoperative treatments.^[14]^ Besides, it has been documented that radiotherapy is feasible, especially for postoperative residual tumors.^[15,16]^ In this case, the tumor was surgically removed, and the lesion did not change significantly during the postoperative follow-up. The patient’s symptoms were remarkably alleviated; therefore, no other adjuvant therapy was administered, except for close follow-up.

## 4. Strengths and limitations

Our study reports a rare case of intraspinal NET and proposes surgical resection as a potential treatment. However, this study had some limitations. However, no known evidence has focused on the risk factors and prognosis of primary intraspinal NETs. Thus, with the advancement of NET treatment, further studies are needed to verify our results and gradually improve the prognosis of intraspinal NETs.

## 5. Conclusions

Because intraspinal NETs are relatively rare, as well as nonspecific symptoms, misdiagnosis and missed diagnosis remain common in clinical settings. In addition to common lumbar neuromuscular diseases, lumbar MRI should be performed in time to exclude the possibility of lumbar NETs, and surgical resection can be an effective treatment for intraspinal NETs.

## Author contributions

**Conceptualization:** Li Dai, Ming-Ju Zou, Bing-Ran Zhang, Ming-Wei Liu.

**Data curation:** Li Dai, Ren-Li Liao, Bing-Ran Zhang, Ming-Wei Liu.

**Funding acquisition:** Li Dai, Zhi-Qiang Ma, Ming-Wei Liu.

**Investigation:** Li Dai, Ming-Ju Zou, Bing-Ran Zhang, Zhi-Qiang Ma, Ming-Wei Liu.

**Methodology:** Li Dai, Zhi-Qiang Ma, Ming-Wei Liu.

**Software:** Li Dai, Ming-Ju Zou, Bing-Ran Zhang, Zhi-Qiang Ma.

**Validation:** Li Dai, Ming-Ju Zou, Ren-Li Liao, Bing-Ran Zhang, Zhi-Qiang Ma.

**Writing – original draft:** Li Dai, Ming-Wei Liu.

**Formal analysis:** Ming-Ju Zou, Ren-Li Liao, Zhi-Qiang Ma.

**Supervision:** Ming-Ju Zou, Ming-Wei Liu.

**Visualization:** Ming-Ju Zou, Zhi-Qiang Ma.

**Resources:** Ren-Li Liao.

**Writing – review & editing:** Ren-Li Liao, Ming-Wei Liu.
